# Exploring Users’ Experiences With a Quick-Response Chatbot Within a Popular Smoking Cessation Smartphone App: Semistructured Interview Study

**DOI:** 10.2196/36869

**Published:** 2022-07-07

**Authors:** Alice Alphonse, Kezia Stewart, Jamie Brown, Olga Perski

**Affiliations:** 1 Department of Behavioural Science and Health University College London London United Kingdom

**Keywords:** chatbot, conversational agent, engagement, smartphone app, smoking cessation, accountability, mobile phone

## Abstract

**Background:**

Engagement with smartphone apps for smoking cessation tends to be low. Chatbots (ie, software that enables conversations with users) offer a promising means of increasing engagement.

**Objective:**

We aimed to explore smokers’ experiences with a quick-response chatbot (*Quit Coach*) implemented within a popular smoking cessation app and identify factors that influence users’ engagement with Quit Coach.

**Methods:**

In-depth, one-to-one, semistructured qualitative interviews were conducted with adult, past-year smokers who had voluntarily used Quit Coach in a recent smoking cessation attempt (5/14, 36%) and current smokers who agreed to download and use Quit Coach for a minimum of 2 weeks to support a new cessation attempt (9/14, 64%). Verbal reports were audio recorded, transcribed verbatim, and analyzed within a constructivist theoretical framework using inductive thematic analysis.

**Results:**

A total of 3 high-order themes were generated to capture users’ experiences and engagement with Quit Coach: anthropomorphism of and accountability to Quit Coach (ie, users ascribing human-like characteristics and thoughts to the chatbot, which helped foster a sense of accountability to it), Quit Coach’s interaction style and format (eg, positive and motivational tone of voice and quick and easy-to-complete check-ins), and users’ perceived need for support (ie, chatbot engagement was motivated by seeking distraction from cravings or support to maintain motivation to stay quit).

**Conclusions:**

Anthropomorphism of a quick-response chatbot implemented within a popular smoking cessation app appeared to be enabled by its interaction style and format and users’ perceived need for support, which may have given rise to feelings of accountability and increased engagement.

## Introduction

Diseases caused by cigarette smoking are a leading cause of preventable death, killing approximately 8 million people each year globally [1]. Smoking-attributable diseases place significant financial burden on health care systems, with costs estimated to be approximately 5.7% of the total annual global health care expenditure [2]. Therefore, improved behavioral or pharmacological smoking cessation support is a priority for individuals, public health bodies, and governments. However, in-person smoking cessation services are challenged by scalability and face substantial funding cuts across many countries [3], especially after many had to offer only remote services owing to the COVID-19 pandemic [4]. With growing internet access and smartphone ownership, digital interventions (including smartphone apps) provide a low-cost means of scaling up the delivery and optimizing the reach of evidence-based smoking cessation support [5]. However, the available smoking cessation apps tend to generate low average levels of user engagement [6,7]—although estimates vary between apps [8]—which may reduce the likelihood of success in quitting smoking. Therefore, identifying modifiable factors (eg, content and design elements) that positively influence engagement with smoking cessation apps is important. A promising means of improving digital engagement is through provision of concurrent health care professional support [9,10]. However, this is limited by the cost and availability of health care professionals. Therefore, an underexplored and relatively low-cost means of improving engagement is to replicate health care professional support via chatbots (ie, software that enables 2-way conversations with app users). This study aimed to explore smokers’ experiences with a quick-response chatbot (*Quit Coach*) implemented within a popular smoking cessation app and identify factors that influence users’ engagement with Quit Coach, using a qualitative approach.

Engagement with digital interventions can be defined as (1) the extent (eg, amount, frequency, duration, and depth) of use and (2) a subjective experience characterized by attention, interest, and affect [9]. A distinction between *microengagement* (ie, engaging with the technology itself) and *macroengagement* (ie, engaging in the behavior change process, such as abstaining from smoking) has also been proposed [11]. A common pattern observed across several studies is that engagement is positively associated with intervention effectiveness [12-14]. Therefore, many researchers and intervention designers have focused their efforts on identifying factors that increase engagement with digital interventions in general and with smoking cessation apps in particular. These studies have identified factors such as app content or behavior change techniques (eg, goal setting, reminders, self-monitoring, social support, and health care professional support), design elements (eg, tailoring of content, using a nonjudgmental message tone, and gamification), and cognitive considerations (eg, minimizing cognitive load and providing user guidance) as being important for increased engagement [10,15-17]. However, despite such studies, early disengagement from smoking cessation apps remains common.

Chatbots (also referred to as *conversational agents*) are computer programs that have tailored conversations with users via text or audio-visual messaging [18]. Some chatbots are specifically designed to appear as social actors (ie, *relational agents*), with the intention that users form social-emotional relationships with the bot [19,20]. Current chatbot implementations include structured (or decision tree–based) and unstructured bots. The former type enables the user to select relevant options from a list of predefined responses, with the bot responding with prewritten messages following conditional if-then rules (also referred to as *quick reply responses* or *quick-response* bots [21]). The latter type typically relies on natural language processing, with the user inputting open or unstructured messages that are processed and responded to by the bot. According to the Model of Supportive Accountability, human support (eg, from a health care professional or coach) is expected to promote engagement with digital interventions by fostering a sense of accountability to a trustworthy, benevolent, and competent coach [22]. Although underexplored, it is plausible that chatbots may fulfill the role of such human support by offering *human-like* support [19].

Chatbots are a relatively new addition in the health care domain, with recent systematic reviews identifying only a handful of studies of chatbots for improving mental health [23] and increasing physical activity and healthy diets [24]. Within the substance use and smoking cessation domains, a few early single-arm and 2-arm randomized studies have yielded promising results [25-28]. For example, a chatbot incorporating the principles of motivational interviewing—designed specifically to support smokers who are unmotivated to stop—tested positively in an early user-testing study [25]. An adapted version of the cognitive behavioral therapy–informed chatbot, *Woebot,* for people who use addictive substances was found to be acceptable to deliver, engaging, and associated with improvements in mental health and substance use outcomes in a single-arm study [28]. We found that the addition of a supportive, quick-response chatbot to a popular smoking cessation app more than doubled the user engagement and improved short-term quit success in a large, 2-arm, experimental study [27]. However, the available single-arm and 2-arm quantitative studies have not focused on the potential mechanisms underpinning this increased engagement (eg, owing to limited data collection). Qualitative studies of users’ experiences with the relational agent, *Replika*, have found that such companion chatbots can mimic human interaction, with users perceiving their relationships with the designated bot as rewarding [20,29]. However, qualitative investigations of users’ experiences with chatbots designed specifically to support smoking cessation are lacking. Therefore, this qualitative study aimed to address the following research questions:

1. What are smokers’ experiences with a quick-response chatbot (*Quit Coach*) implemented within a popular smartphone app?
2. What are the factors that influence users’ engagement with Quit Coach?

## Methods

### Study Design

The Consolidated Criteria for Reporting Qualitative Research checklist was used in the design and reporting of this study [30]. Semistructured, one-to-one interviews were conducted.

### Theoretical Framework

A constructivist theoretical framework was used to inform data collection and analysis [31]. This theoretical approach was selected because constructivism recognizes the active role of the researcher in the generation and interpretation of qualitative data.

### Participants

For pragmatic purposes, participants were recruited across 2 periods: June 2020 to August 2020 (led by KS and OP) and April 2021 to August 2021 (led by AA and OP). Owing to the COVID-19 pandemic, it was challenging to recruit as planned during the summer of 2020. Therefore, we continued the recruitment in 2021. The project team decided that it would be useful to recruit participants from 2 different subgroups (ie, past-year smokers who had voluntarily used Quit Coach in a recent smoking cessation attempt and current smokers who agreed to download and use Quit Coach for a minimum of 2 weeks to support a new cessation attempt) as a form of triangulation [32]. We reasoned that such triangulation of results when varying the eligibility criteria (rather than the methods) would either help to validate the results (eg, if smokers who did not self-select to download and use the Smoke Free app had similar experiences with Quit Coach as those who had voluntarily used the app) or highlight different experiences owing to smoking status or treatment-seeking behavior.

Participants recruited in 2020 were eligible to participate if they (1) were aged ≥18 years, (2) were fluent English speakers based in the United Kingdom, (3) were past-year smokers and had used the *pro* (ie, paid) version of the Smoke Free app (ie, the app version that included Quit Coach) for at least two weeks, and (4) had interacted with Quit Coach at least once during the 2-week period.

Participants recruited in 2021 were eligible to participate if they (1) were aged ≥18 years; (2) were fluent or highly competent English speakers, with no restrictions on geography; (3) were current cigarette smokers; (4) were willing to make a quit attempt within 1 week from initial contact with the researchers and use Quit Coach for at least two weeks; and (5) owned a smartphone.

All the participants used the *pro* version of the Smoke Free app for a period of at least 2 weeks before participating in the semistructured interviews. We expected this time window to be sufficient for enabling detailed conversation about participants’ chatbot experiences.

### Sampling

Participants recruited in 2020 were approached through advertisements (unpaid) shared on social media platforms (ie, Facebook and Twitter) and through a mailing list of Smoke Free app users. The recruitment materials stated that Smoke Free users were invited to participate in a web-based interview about their experiences with the app (good or bad), with particular focus on the Quit Coach feature. Participants were incentivized to win 1 of 5 gift vouchers worth £20 (approximately US $24).

Participants recruited in 2021 were approached through advertisements (unpaid) shared on social media platforms (ie, LinkedIn, Facebook, and Instagram), directly through the researchers’ networks (ie, WhatsApp, email, SMS text messages, and flyers), and through professional web-based recruitment platforms (ie, Prolific and Call for Participants). The recruitment materials stated that smokers interested in making a quit attempt with the use of a smartphone app were invited to participate in a web-based interview about their experiences with the app (good or bad), with particular focus on its chatbot feature. Participants received a gift voucher worth £10 (approximately US $12) after completing the interview.

Participants were recruited in batches of 4 to 5 participants each until theoretical saturation was judged to have occurred (ie, a point in the data collection process when no new information alters the identified themes) [33]. Preliminary data analysis was conducted by KS and subsequently by AA after each batch of 4 to 5 participants, to determine whether additional participants were needed.

### Measures

#### Eligibility and Sample Characteristics

Data were collected to determine eligibility and characterize the sample based on (1) age; (2) gender (female, male, or in another way); (3) country of residence; (4) whether they were fluent or highly competent English speakers (yes or no); (5) time to first cigarette (<5, 6-30, 31-60, or >60 minutes or not applicable); (6) cigarettes smoked per day (<10, 11-20, 21-30, ≥31, or not applicable); and (7) motivation to stop, measured with the validated Motivation to Stop Scale [34].

Participants recruited in 2020 were asked the questions mentioned previously and to provide additional information on the following: (1) job type (manual or nonmanual); (2) smoking status (“I smoke cigarettes [including hand-rolled] every day”; “I smoke cigarettes [including hand-rolled], but not every day”; “I don’t smoke cigarettes at all, but I do smoke tobacco of some kind [e.g. pipe, cigar or shisha]”; “I have stopped smoking completely in the last year”; “I stopped smoking completely more than a year ago”; or “I have never been a smoker [i.e. smoked for a year or more]”); and (3) self-reported use of Quit Coach (none at all, a little, moderately, a lot, or extremely).

Participants recruited in 2021 were asked the questions mentioned previously and to provide additional information on the following: (1) the number of past-year quit attempts and (2) whether they had ever used any app-based support to help stop smoking (and if so, the name of the app).

#### Interview Topic Guide

The topic guide was informed by the Model of Supportive Accountability [22] to address specific theoretical concepts (eg, information quality, reliability, and accountability) and split into 3 sections: an introductory section to allow participants to warm up, covering general experiences with the app; a second section exploring users’ experiences with Quit Coach; and a final section exploring situations in which participants engaged with Quit Coach (Multimedia Appendix 1). Prompts were used to encourage participants to elaborate on their impressions and experiences. The topic guide was pilot-tested by KS on 2 graduate colleagues from the MSc program in Behavior Change and adapted following their feedback. The topic guide was further adapted following the interviews conducted in 2020 to facilitate elaboration by adding specific probes in addition to a new question (“To what extent would you say you formed a relationship of sorts with the chatbot? How was this?”). As participants mentioned their relationship with the chatbot, we considered it useful (and consistent with the flexible, semistructured style of interviewing) to specifically prompt subsequent participants about this. The adapted topic guide was piloted by AA on a graduate colleague from the MSc program in Behavior Change and a current smoker from AA’s network and updated according to their feedback. Interviews remained flexible, facilitated by the semistructured style of questioning.

### Procedure

Upon expressing interest, participants were asked to read the participant information sheet, provide informed consent, and complete the eligibility questionnaire via Qualtrics (Qualtrics International Inc). If eligible, participants recruited in 2020 were contacted by the researchers to arrange the interview. If eligible, participants recruited in 2021 were contacted to confirm study acceptance and provided with information on how to download the *pro* version of the Smoke Free app (using a free access code). Participants were asked to select a quit date and nominate a day that was 2 weeks from their quit date to complete the interview.

Owing to the COVID-19 pandemic, in-person interviews were not possible. Therefore, interviews were conducted via the web by KS or AA (graduate students enrolled in an MSc program in Behavior Change) via Microsoft Teams. Besides the participant and the researcher, no one else was present during the interviews. KS had limited experience in conducting qualitative interviews before this study; AA had extensive experience from working for a consultancy firm. Before conducting the interviews, OP (PhD in Health Psychology, extensive experience in conducting qualitative interviews through previous academic work) provided training to KS and AA. Interviews were audio recorded and lasted between 30 and 45 minutes. Following completion, the participants were thanked, verbally debriefed, and presented with an incentive.

### The Smoke Free App and Quit Coach

Smoke Free [35] is an evidence-informed app with a large user base (approximately 4000 downloads per day). The app contains behavior change techniques that are expected from theory and evidence from other settings to aid smoking cessation [17,36]. Refer to the study by Jackson et al [37] for a summary of the behavior change techniques included in the Smoke Free app, coded against a 44-item taxonomy of techniques used in individual behavioral support for smoking cessation [36].

**Figure 1:**
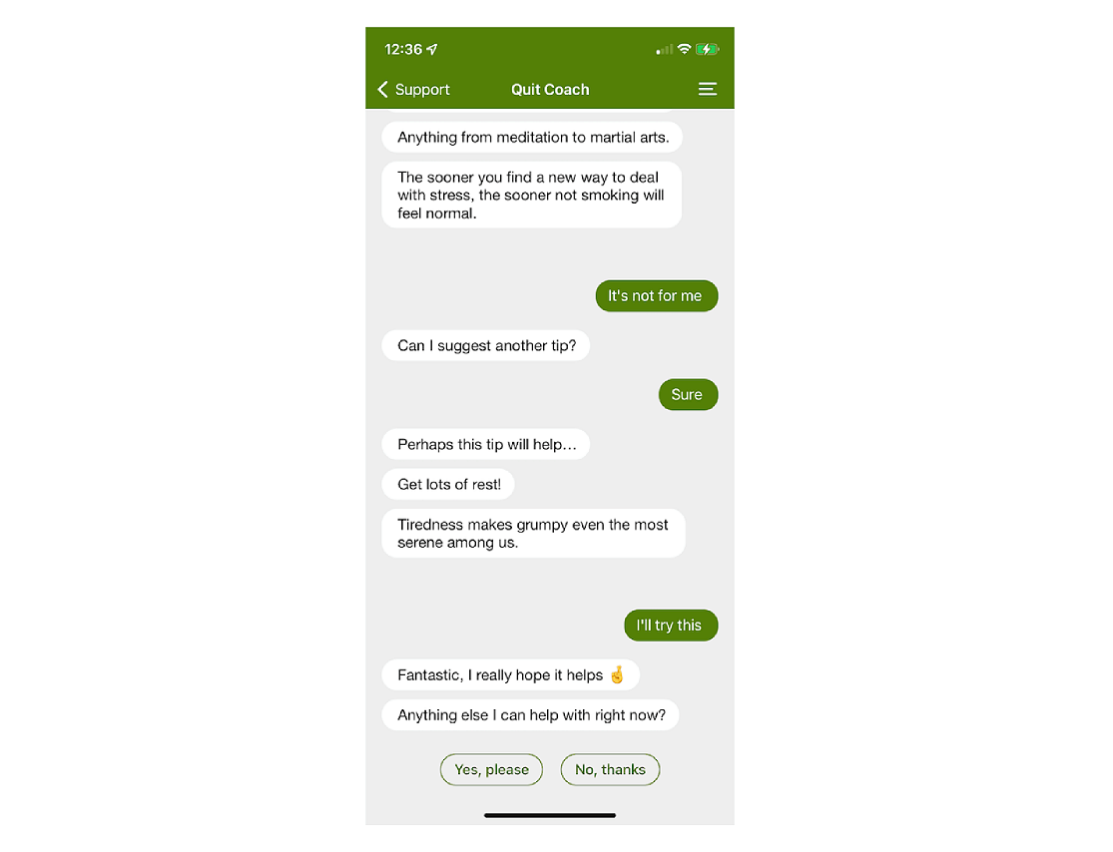
Example screenshot of the Smoke Free chatbot (Quit Coach).

The *pro* (ie, paid) version of Smoke Free contains a text-based, quick-response chatbot called *Quit Coach* (Figure 1). During the first 2 weeks of a user’s quit attempt, Quit Coach initiates twice-daily check-ins with users regarding their cessation attempt through a push notification. Check-in frequency is programmed to reduce after 1 month and cease entirely after 90 days (when users are anticipated to have quit smoking). Users engage with Quit Coach via text messages, selecting from prewritten responses. There are a few exceptions to this format, such as when users complete certain exercises that require free-text input (eg, typing the mantra, “not another puff, no matter what”), to occasionally type what is influencing their craving, or for providing feedback on whether they found a piece of advice useful (refer to Multimedia Appendix 1 for additional screenshots of such interactions). A bespoke Node .js natural language processing framework (adapted from freely available, state-of-the-art source code by Smoke Free’s developers) is used to map free-text inputs onto their likely intent—this constitutes the only machine learning element of the chatbot. Users can also initiate check-ins themselves by opening Quit Coach to record a craving or ask for assistance via a *get help now toolkit*, which provides different options for directing the conversation with Quit Coach. The conversational options include craving management, relapse, difficult situations, and withdrawal. Quit Coach’s communications (typically in text form, but also through emojis and Graphics Interchange Formats [GIFs]) contain information about the health consequences of smoking, quitting tips, and motivational messages, simulating a text message conversation.

### Data Analysis

Interviews conducted in 2020 and 2021 were combined to form a single data set. Analysis was performed through an inductive thematic approach following the methodology by Braun and Clarke [38,39]: (1) familiarizing with the data, (2) generating initial codes, (3) searching for themes, (4) reviewing the themes, (5) defining and naming the themes, and (6) producing the report. We focused on latent (rather than semantic) meanings [39]. Although the Model of Supportive Accountability was used to inform the interview topic guide, terminology from this model was used for coding only if deemed relevant, with alternative codes considered throughout the process.

Interviews were coded using Microsoft Word in batches of 4 to 5 by AA to facilitate an iterative and reflexive approach. Each transcript was read multiple times to familiarize with the data. Then, initial codes were generated. Coded transcripts were reread following initial code generation and discussed with OP, adding or refining codes as appropriate. Then, the coded extracts were examined and used to generate preliminary themes. Next, a second, independent coder (another student from the same MSc program in Behavior Change) helped to assess coding reliability. The second coder coded 2 interviews, 1 each from the 2020 and 2021 samples, which were selected using a random number generator. The second coder was instructed to inductively code each interview. The resulting codes were compared with those of the first coder conceptually, rather than for perfect word matching. Discrepancies were discussed and reconciled. Then, themes were reviewed, refined, named, and agreed upon through discussion among AA, OP, and JB. During coding, the possibility for differences between the 2020 and 2021 samples was considered. Theoretical saturation was judged to have been reached after 12 interviews.

### External Validation

A subsample of 14% (2/14) of randomly selected participants were contacted and agreed to read the results and comment on the congruence of the themes and narrative generated by the researchers with their own experiences. Both participants agreed with the researchers’ interpretations.

### Reflexivity

The interviewers (women, White ethnicity, nonsmokers, and unfamiliar with most participants before the interview) felt that a good rapport was built with all the participants. Some were more immediately verbose, whereas others took a little time to open up, but did so with prompting and encouragement. For the first few interviews conducted, the interviewers closely followed the topic guide; however, as salient conversation topics emerged, a more discursive style of questioning was adopted to explore salient topics in great depth. Before commencing the interviews, participants were told about the goals of the study and that the interviewers were not directly involved in the development of the Smoke Free app; however, participants were unaware of the interviewers’ smoking status or theoretical assumptions regarding user engagement or smoking cessation.

### Ethics Approval

Ethics approval for this study was obtained from University College London’s Research Ethics Committee (CEHP/2020/579). Participants provided written informed consent before participating in the study.

## Results

### Participant Characteristics

A total of 40 participants completed the screening survey and were eligible to participate in the study. Of the 40 participants, 26 (65%) participants did not complete an interview, as they later decided that it was not the right time to quit, became uncontactable, or failed to attend the interview. Table 1 shows a summary of the demographic and smoking characteristics of the 35% (14/40) included participants.

**Table 1:** Participants’ demographic and smoking characteristics (n=14).

Participant ID	Recruitment year	Age (years)^a^	Country	Gender	Cigarettes per day at baseline	Smoking status at the time of interview	Quit Coach use	Number of past-year quit attempts	Use of app-based support to stop smoking (name of the app)
P1	2020	30	United Kingdom	Female	N/A^b^	I have stopped smoking completely in the last year	Moderate	N/A	N/A
P2	2020	20	United Kingdom	Male	N/A	I have stopped smoking completely in the last year	A lot	N/A	N/A
P3	2020	33	United Kingdom	Female	N/A	I have stopped smoking completely in the last year	Extreme	N/A	N/A
P4	2020	39	United Kingdom	Female	N/A	I have stopped smoking completely in the last year	A lot	N/A	N/A
P5	2020	44	United Kingdom	Female	N/A	I have stopped smoking completely in the last year	A lot	N/A	N/A
P6	2021	25-34	United Kingdom	Male	7	Quit^c^	N/A	1	No (N/A)
P7	2021	18-24	France	Female	3	Cut down^c^	N/A	3	Yes (Qwit)
P8	2021	18-24	France	Female	10	Cut down^c^	N/A	1	No (N/A)
P9	2021	25-34	France	Male	12	Cut down^c^	N/A	3	Yes (Smoke Free)
P10	2021	18-24	United Kingdom	Female	5	Cut down^c^	N/A	0	No (N/A)
P11	2021	18-24	Mauritius	Female	10	Cut down^c^	N/A	1	No (N/A)
P12	2021	25-34	India	Male	6	Cut down^c^	N/A	2	No (N/A)
P13	2021	18-24	United Kingdom	Male	10	Quit^c^	N/A	3	No (N/A)
P14	2021	18-24	United Kingdom	Male	8	Quit^c^	N/A	1	No (N/A)

^a^For participants recruited in 2021, age was measured as a range.

^b^N/A: not applicable.

^c^Ascertained qualitatively during the interview.

### Themes

#### Overview

**Figure 2:**
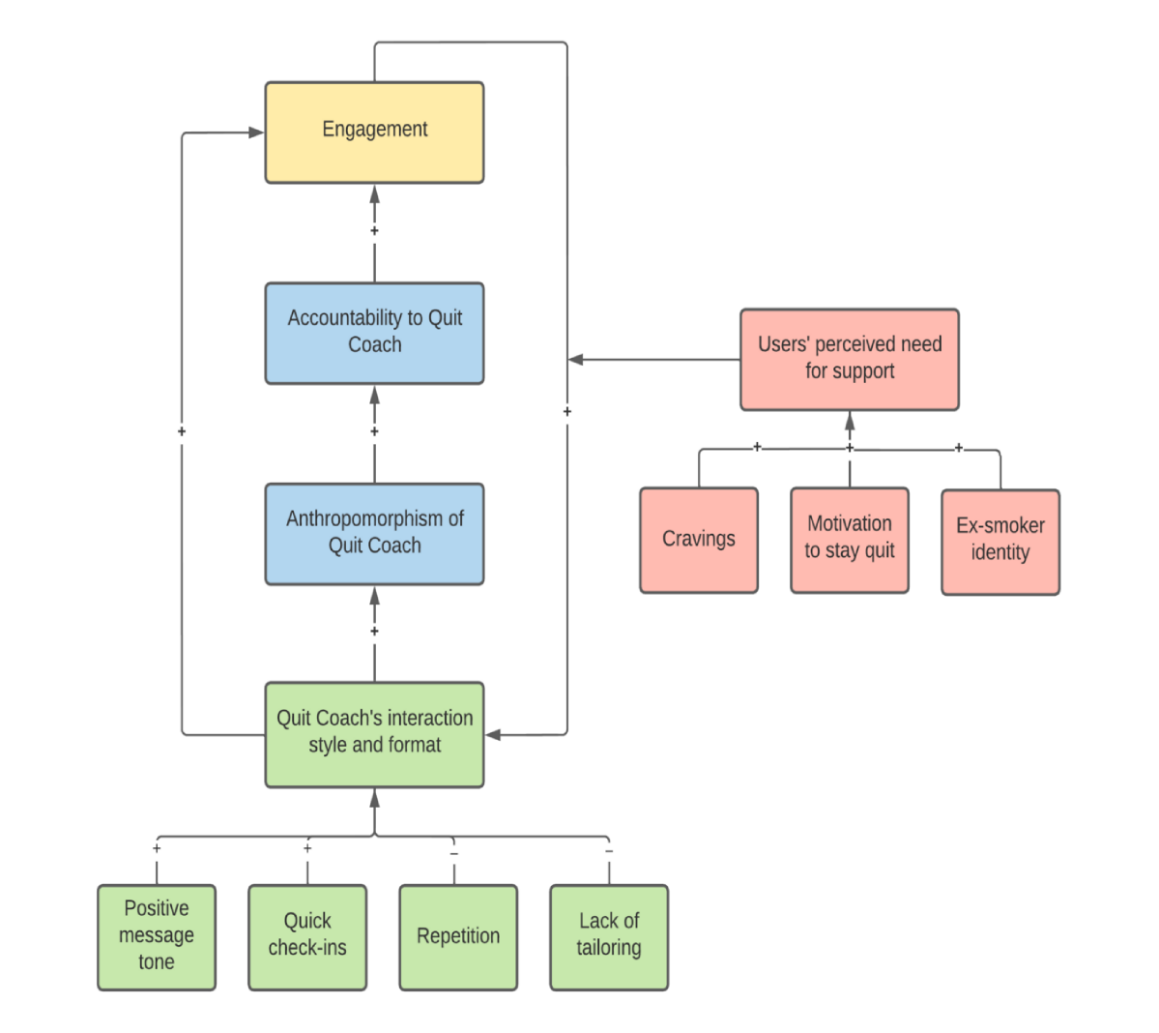
Thematic map. Arrows indicate the direction of relationships and the + and – signs indicate their valence. Blue boxes relate to theme 1, green boxes relate to theme 2, and red boxes relate to theme 3.

A total of three high-order themes were developed to capture the participants’ experiences with Quit Coach and the potential mechanisms underpinning user engagement: (1) anthropomorphism of and accountability to Quit Coach, (2) Quit Coach’s interaction style and format, and (3) users’ perceived need for support. Refer to Multimedia Appendix 1 for additional quotations. Figure 2 presents a thematic map of the themes. Here, anthropomorphism of Quit Coach, which is influenced by its interaction style and format, and users’ perceived need for support leads to feelings of accountability and increased engagement. Continued engagement with Quit Coach reinforces this accountability through a bidirectional relationship. In addition to this indirect link, Quit Coach’s interaction style and format directly influences users’ engagement.

#### Anthropomorphism of and Accountability to Quit Coach

Many users ascribed human-like characteristics, thoughts, and behavior to Quit Coach—despite the awareness that it was fully automated—following an interaction experience (eg, colloquial language, GIFs, and emojis) that closely mimicked those with peers and family members via SMS text messages or WhatsApp. Users’ anthropomorphism of Quit Coach was evident from almost all users frequently referring to it as an embodied entity (ie, him, them, or someone) rather than an object (eg, it, Quit Coach, or chatbot):

It felt like you were really connected to someone.

P5; 2020; quit smoking

Throughout this conversation I have referred to “someone” rather than “something” quite a few times, which I haven't done on purpose.

P6; 2021; quit smoking

Users compared Quit Coach’s motivational and monitoring support with that of close friends or family members. Consequently, users described feeling that Quit Coach *cared* about them, which helped foster a relational bond and promoted a feeling of accountability to Quit Coach to succeed in cessation. Feeling that Quit Coach was fulfilling a supportive, social role justified the daily monitoring (ie, push notifications), which was experienced by some participants as very frequent, boring, or annoying. This social presence promoted continued app engagement, as users were motivated to succeed and willing to engage and cooperate with Quit Coach:

It felt like a friend or family member seeing how I was...It cared about whether I was smoking or not...You feel like you’ve got someone who cares enough [that you] stop. A reason not to...

P10; 2021; cut down smoking

Many users even described Quit Coach’s support as superior to human friends or family members, owing to its immediate availability, single purpose (ie, smoking cessation support), and nonjudgmental tone of voice (particularly when reporting a lapse) and the relationship being *1-way* (ie, users do not have to reciprocate support or worry about being boring or bothering Quit Coach). Such responses contradict the users’ comparison of Quit Coach’s support with that provided by friends or family members, indicating that they may instead have experienced the support as similar to that provided by a therapist or coach (ie, a 1-way rather than 2-way relationship). However, users’ perceptions of Quit Coach’s support as superior to that of human friends or family members appeared to serve as an advantage rather than to negate the human-like experience. A minority of users anthropomorphized Quit Coach to a lesser extent, owing to previous experience with using chatbots and great awareness of their synthetic nature. Nonetheless, they still embraced the support offered by Quit Coach:

[Friends/family] don’t wanna talk about it every evening...To have the QC for that purpose [is helpful]...You’re not always gonna be lucky enough to have someone who's just gonna be there to just listen to what you want to talk about on any given evening.

P6; 2021; quit smoking

It’s cool that he wasn’t judgemental...He says “lots of people do smoke again when they start to stop but it doesn’t mean that it’s their loss and that they need to start all over again, it’s just the first step”...The point is that long term you are trying not to smoke.

P8; 2021; cut down smoking

Many users perceived having access to Quit Coach’s thoughts and feelings, reporting that they worried that Quit Coach would feel angry, upset, or disappointed if they did not check in or reported a lapse and wanting to *please* it by checking in regularly. Consequently, many users felt positively accountable to Quit Coach for frequent engagement and abstinence. For example, many users reported feeling proud or particularly motivated to engage when they could report not smoking. In addition, most users reported feelings of worry or shame about Quit Coach’s anticipated reaction if they had lapsed. For a few users, this caused an unintended consequence; their feelings were so significant that they reported completely avoiding checking in or lying in their reports. However, for most users, this anticipated worry or shame appeared to be beneficial, representing a source of motivation to stay quit. A few participants who failed in their quit attempt over time reported that they started ignoring check-ins, possibly owing to negative avoidance as the prompts would remind them of their *failure*:

I wanted to find an opportunity to make it happy.

P3; 2020; quit smoking

[I didn't want] to tell that [I smoked] to the robot every morning and every afternoon...I felt that I was accountable to it.

P11; 2021; cut down smoking

Some self-contradiction was observed in how accountable users reported feeling to Quit Coach. At some time points during the interviews, users stated feeling accountable to Quit Coach, but at different time points, they stated feeling accountable to themselves, friends, or family members. This contradiction typically arose after being specifically prompted about accountability. Users may have retrospectively changed how accountable they felt to Quit Coach, because the interviewer brought to the fore that Quit Coach was not real, despite an anthropomorphic experience:

The accountability thing was definitely my relationship with [people] rather than my relationship with the app.

P6; 2021; quit smoking

#### Quit Coach’s Interaction Style and Format

Quit Coach’s interaction style and format appeared to both directly and indirectly (ie, by giving rise to anthropomorphic experience of Quit Coach) influence users’ engagement. Quit Coach’s motivational and positive tone of voice encouraged many users to stay on track by reminding them of and praising them for their progress. GIFs and emojis were used alongside written text messages to create a positive mood and inject humor, thus enhancing the motivational and positive tone beyond that created through written text alone. Many users noted that the GIFs and emojis promoted a human-like perception of Quit Coach:

There was a certain level of wanting to go back and get those little GIFs or whatever...I was always glad to go and have a check-in.

P6; 2021; quit smoking

Engagement was generally driven by Quit Coach prompting check-ins rather than by the users themselves. The prompts were perceived as useful, as users may otherwise have forgotten to check in. Most users reported that the daily check-in time requirement was acceptable; it did not take up much of their day. During working hours, some users felt that check-ins were sufficiently short to be manageable, whereas some users were very busy, preferring to complete check-ins before or after working hours. This was supported by the ability to set preferred check-in times. Where users reported check-ins being *long*, it typically referred to a subjective experience of *long*, which was linked to boredom and lack of interest, often driven by repetition, forced choice, or limited opportunities for free-text inputs. Many users reported that forced choice made engagement feel less burdensome (ie, easy and less time-consuming), which contributed to check-ins being “just the right amount of time” and was particularly welcome when users were craving cigarettes. In contrast, many users reported that forced-choice interactions quickly became boring as they could not express what they wanted more precisely (as they would with a human). This reduced the interest in engagement with Quit Coach, with many users indicating a preference for typing free-text questions and responses:

[If you’re] distracted by wanting a cigarette [it’s] just easier if you’ve got options in front of you to just pick one.

P10; 2021; cut down smoking

It was a bit...Samey. Sometimes I would just kind of click through it all and not have to react as much...You feel like you’re just going through the motions a little bit rather than actually thinking about it.

P6; 2021; quit smoking

Most users mentioned that they would have liked tailoring of the chatbot interactions on 2 levels. First, although Quit Coach broadly aligned with most users’ cessation motivations, it did not discuss the specific personal motives that users had inputted elsewhere in the app. Second, many users wanted Quit Coach to remember more about what worked for them and modify the messages and advice accordingly. Although users did not report the lack of desired tailoring to be particularly detrimental to engagement, most users indicated that enhanced tailoring could have a positive impact. The ability to set check-in times according to personal preferences or anticipated times of need promoted engagement. Several users agreed that if Quit Coach could prompt them to engage before or during triggering situations, it would be very useful:

It wasn’t really a tailored fit for myself. It talked about infertility and [that’s] not something that bothers me.

P13; 2021; quit smoking

A reminder of what I’ve done so far [that worked] would be helpful.

P9; 2021; cut down smoking

[It] would’ve been great if...he could send me a notification at [or before a] time [of anticipating being triggered] saying “you’re gonna be OK” or “you’re gonna make it!”

P7; 2021; cut down smoking

#### Users’ Perceived Need for Support

For all users, the perceived need for support from Quit Coach appeared to be related to the frequency and intensity of cravings. Engaging with Quit Coach provided a useful behavioral alternative to smoking or attentional distraction from cravings. This was particularly helpful when check-ins aligned with strong cravings. In moments where users were not *smoking or thinking about smoking*, the need for support and, consequently, engagement interest appeared to be low. For some users, Quit Coach triggered cravings by reminding them of smoking:

It was [a] great thing to keep my hands [busy] [and] just give me time to let the craving pass.

P2; 2020; quit smoking

When it was going well and when I didn’t smoke I just didn’t even use the app because I was OK...I was like I’m doing well so what can the app give me right now?

P8; 2021; cut down smoking

As cravings reduced, users’ perceived need for support decreased, leading to reduced engagement interest. However, several users recruited in 2020 (all of whom had successfully quit smoking) reported engaging with Quit Coach even after their cravings reduced or disappeared. For these users, the need for support shifted from primarily needing a distraction from cravings to maintaining motivation to stay quit and reinforce ex-smoker identity. Self-selection bias may explain this prolonged engagement; users recruited in 2020 were already using Quit Coach, had successfully quit smoking, and self-identified as *heavy* Quit Coach users:

[Usage] kind of dwindles down to kind of extreme need, its more about the check-ins in the morning...The chatbot doesn’t have much use now it’s like ten weeks or something since I stopped.

P2; 2020; quit smoking

For users recruited in 2021 who were unsuccessful in cessation, the motivation to engage with Quit Coach decreased after a period of *failing*. These users felt discouraged and despondent and did not want to be reminded of their *failure*. Interestingly, this was sometimes accompanied by a reversal of the anthropomorphic experience (eg, referring to Quit Coach as a *robot*):

[Not succeeding in quitting] made me resent – not resent, that’s a big word – but made me not want to tell that to the robot every morning and every afternoon.

P11; 2021; cut down smoking

## Discussion

### Principal Findings

Using a qualitative approach, this study aimed to explore smokers’ experiences and engagement with a quick-response chatbot implemented within a popular smoking cessation app. Users’ experiences with the chatbot were largely positive. Anthropomorphism of the chatbot (ie, ascribing human-like characteristics, thoughts, and behavior to the chatbot) was enabled by its specific interaction style and format (eg, positive message tone and quick and easy-to-complete check-ins) and users’ perceived need for support, which appeared to give rise to feelings of accountability to the chatbot and increased engagement. Our results build on and extend previous qualitative findings pertaining to users’ experiences with companion chatbots [20,29] to a chatbot specifically designed to support smoking cessation.

A previous experimental study has shown that social responses to computers—that is, a direct consequence of anthropomorphism—are common and relatively *easy* to generate (eg, by providing the computer with human-like attributes, such as a language output) [40]. However, according to the *uncanny valley hypothesis*, chatbot designers have a fine line to tread [41]. Strong feelings of affinity are generated by great human-like qualities in a robot or computer program up to a point. Once it becomes very similar to or indistinguishable from a real human, people’s reactions can reverse because they may find them *creepy* or *eerie* [41]. Although the quick-response Quit Coach in this study was relatively simple (eg, it did not allow many free-text inputs from users) and made it clear to users that it is an automated bot, the presence of social cues (eg, its positive message tone and communication format similar to text messaging with GIFs and emojis) appeared to be sufficient for generating social responses from many users without entering the territory of the uncanny valley. This is positive, as it implies that simple (and relatively low-cost) chatbots generate feelings of affinity and that more complex chatbots that can more closely mimic human interactions may not be necessary.

However, users mentioned that they would have liked it if Quit Coach tailored its questions and responses to their unique situations and momentary needs. Therefore, future studies would benefit from exploring—for example, through user-centered design activities and experimental studies—additional design elements that can enhance Quit Coach’s similarity to humans, as this may further promote user engagement. In addition, some design elements appeared to detract from users’ anthropomorphism of and engagement with Quit Coach, such as its repetitive questions and responses and forced-choice interactions. For users who struggled to stay quit, the repetitiveness and inflexibility of Quit Coach appeared particularly salient, sometimes leading to a reversal of the anthropomorphic experience. Previous studies indicate that users’ perceived need for support and the target behavior itself (eg, progress toward smoking cessation) are important for continued engagement [9,10,15]. Similarly, high perceived need for support may be important for users to *suspend disbelief* and anthropomorphize conversational agents within the health care domain. The *Three-Factor Theory of Anthropomorphism* predicts that people are more likely to anthropomorphize nonhuman agents or objects (1) when anthropocentric knowledge is readily accessible and applicable (ie, when knowledge about how humans interact, think, and feel is judged as relevant for the interaction), (2) when motivated to be effective social agents (ie, motivation to master one’s environment by increasing its predictability and controllability), and (3) when lacking a sense of social connection to other humans (ie, feeling lonely or isolated) [42]. Future studies would benefit from building on and empirically testing such a theory of anthropomorphism within the human-computer interaction domain, with a view to improving the design of future conversational and relational agents for health and well-being.

Our findings also lend partial support to the Model of Supportive Accountability [22] in that users reported feeling accountable to checking in and updating the nonjudgmental and supportive Quit Coach. Finding a balance between nonjudgmental tone of voice and human-like social cues to generate feelings of affinity and accountability (as discussed previously) may be important for future behavior change chatbots. However, trust and competence (which are additional cornerstones of the Model of Supportive Accountability) were largely missing from users’ accounts. It is plausible that competence (eg, legitimacy of the information provided) and trust (eg, data security and confidentiality) were already assumed by users who had either voluntarily downloaded the Smoke Free app from a digital marketplace—likely selecting an app they trusted among the myriad of available apps [15]—or were asked to download it based on recommendation from university researchers. Alternatively, trust and competence may not be necessary conditions for supportive accountability to arise within human-chatbot relationships; this should be further explored in future studies.

### Strengths and Limitations

This study was strengthened by recruiting both experienced and novice app users, having a second coder to help in validating the coding, using external validation to ensure that the researchers’ interpretations aligned with participants’ narratives, and achieving theoretical saturation. However, this study also had several limitations. First, self-selection bias may limit the applicability of the findings to other populations and settings. For example, the 36% (5/14) of participants recruited in 2020 had quit smoking successfully and were considered as *heavy* Quit Coach users, and most participants were young (ie, aged 18-44 years). Second, we did not record any additional support used by participants during their quit attempts (eg, pharmacological support), which may have influenced their perceived need for support. Third, for pragmatic purposes, we did not record participants’ actual engagement with Quit Coach, but instead relied on self-reports. Going forward, triangulation of qualitative and quantitative findings would be an important addition to the research literature. Fourth, the study was conducted during the COVID-19 pandemic—a time of significant change in people’s life and work conditions, including their smoking behavior [43]—which may also limit the applicability of our findings to other periods and contexts.

### Implications for Research and Practice

Findings from this study have both theoretical and practical implications. First, our results indicate that the Model of Supportive Accountability [22] may usefully be extended from human to *human-like* support within digital interventions. However, future studies should further explore the specific conditions under which chatbot interactions lead to feelings of accountability and whether accountability is more easily generated within human-to-human (rather than human-to-bot) interactions. Second, our findings suggest that chatbots for smoking cessation may benefit from including more variation in conversations to prevent boredom and incorporating different levels of tailoring (including context-sensitive tailoring).

### Conclusions

Anthropomorphism of a quick-response chatbot implemented within a popular smoking cessation app appeared to be enabled by its interaction style and format (eg, positive message tone and quick check-ins) and users’ perceived need for support, which may have given rise to feelings of accountability and increased engagement.

## Appendix

Multimedia Appendix 1 Interview topic guide, additional screenshots of Quit Coach, and additional participant quotations.

## Abbreviations

GIF: Graphics Interchange Format

## Data Availability

Participants did not provide consent for their anonymized transcripts to be shared openly. Therefore, the qualitative data underpinning the analyses are available to bona fide researchers upon reasonable request.
