# Supplementary material for: Exploring Users’ Experiences With a Quick-Response Chatbot Within a Popular Smoking Cessation Smartphone App: Semistructured Interview Study
Source: JMIR Form Res. 2022 Jul 7;6(7):e36869. doi: 10.2196/36869 (PMC9305398; doi:10.2196/36869)
Supplement: Multimedia Appendix 1 [file formative_v6i7e36869_app1.docx]

**Supplementary Materials**

Interview topic guide – 2020

I’m going to start with some more general questions:

1. Can you tell me a bit about your overall experience using the Smoke Free app?
   1. Were there any aspects you particularly liked? Why?
   2. Were there any aspects you particularly disliked? Why?

Now, I’ve got a few specific questions about the “Quit Coach” feature, which was the chatbot feature available under the ‘support’ tab of the app, I’ll refer to it as “Quit Coach” as I go through the questions. So, do you know which feature I am talking about?

1. Just in your own words, can you describe how you found using the Quit Coach?
   1. Why do you think you found that?
2. Can you tell me a bit about the way the Quit Coach interacts with you?
   1. What kind of style of the conversation did the chatbot have with you?
   2. How did you find this?
   3. Why do you think you found that?
3. How did you find being asked to “check in” by the Quit Coach? This was through notifications asking you things like “how are you doing?” at set times a day.
   1. How did you find the responses it gave to your check-ins?
   2. Why do you think you feel it was like that?
4. How did you find the quality of the information the Quit Coach gave you about quitting smoking? By ‘quality’ I mean the degree to which you think it was
   1. Why do you think you felt like that?
5. How technically reliable did you find the Quit Coach?
   1. That is interesting, can you expand more on that?
6. How accountable did you feel towards the Quit Coach? By ‘accountable’ I mean how much you felt required or expected to justify your actions or decisions to the chatbot.
   1. Why do you think you felt this way?
7. Do you feel the chatbot had a specific purpose? If so, what do you think it was?
   1. Why do you think you felt that was the case?
   2. How effectively do you think the chatbot succeed in its purpose? Why?
8. Do you feel the chatbot was aligned with your goals/motivations for wanting to stop smoking?
   1. Why/why not?
   2. When you talk about X, it would be good if you could expand on that?
9. Do you remember what situations you used the Quit Coach in?
   1. Why was that?
   2. Where there any times that you would not interact with it or would ignore it? Why was that?
10. Overall, is there anything else like to add about the experience interacting with the Quit Coach? Please take your time to consider.

Interview topic guide – 2021

1. Can you tell me a bit about your overall experience using the Smoke Free app?
   1. Were there any aspects you particularly liked? Why?
   2. Were there any aspects you particularly disliked? Why?
   3. If respondent not chatty - Please could you summarise your overall experience in 2-3 words. Probe for rationale of word choice.

Now, I’ve got a few specific questions about the chatbot/Quit Coach feature.

1. Just in your own words, can you describe how you found the chatbot?
   1. Why?
2. Can you tell me a bit about the way the chatbot interacts with you?
   1. What kind of style of the conversation did the chatbot have with you?
   2. How did you find this?
   3. Why do you think you found that?
3. How did you find being asked to “check-in”?
   1. If not covered organically: To what extent were the check-ins manageable?
   2. How did you find the responses it gave to your check-ins?
   3. If relevant – Specifically, how did you find responses when you checked in reporting that you had lapsed vs. when you had not smoked?
   4. How did you feel after check-ins with the Quit Coach, whether they had been reporting progress or a lapse?
4. How did you find the quality of the information the Quit Coach gave you? [quality = reliable, believable, trustworthy]
   1. Why?
   2. Would you have liked to know more about anything in particular?
   3. What resonated the most?
5. How reliable was your experience of the chatbot? [reliable = consistency in responses, information it gave you, technical reliability]
   1. Can you expand on that?
   2. Were there any ways it was un/reliable?
6. How accountable did you feel towards the Quit Coach? [accountable = how much you felt required or expected to justify your actions or decisions to the chatbot]
   1. Why?
7. To what extent would you say you formed a relationship of sorts with the chatbot?
   1. How was this?
8. Do you feel the chatbot had a specific purpose? If so, what do you think it was?
   1. What made you think that?
   2. How effectively do you think the chatbot succeeded in its purpose? Why?
9. Do you feel the chatbot was aligned with your goals/motivations for wanting to stop smoking?
   1. Why/why not?
10. Do you remember what situations you used the chatbot in?
    1. Were any more/less likely than others?
    2. Where there any times in particular that you wouldn’t interact with it or ignore it? Why?
11. Overall, is there anything else you would like to add about what aspects could be changed or kept, to improve the chatbot experience?

Additional screenshots of the Smoke Free chatbot (‘Quit Coach’)


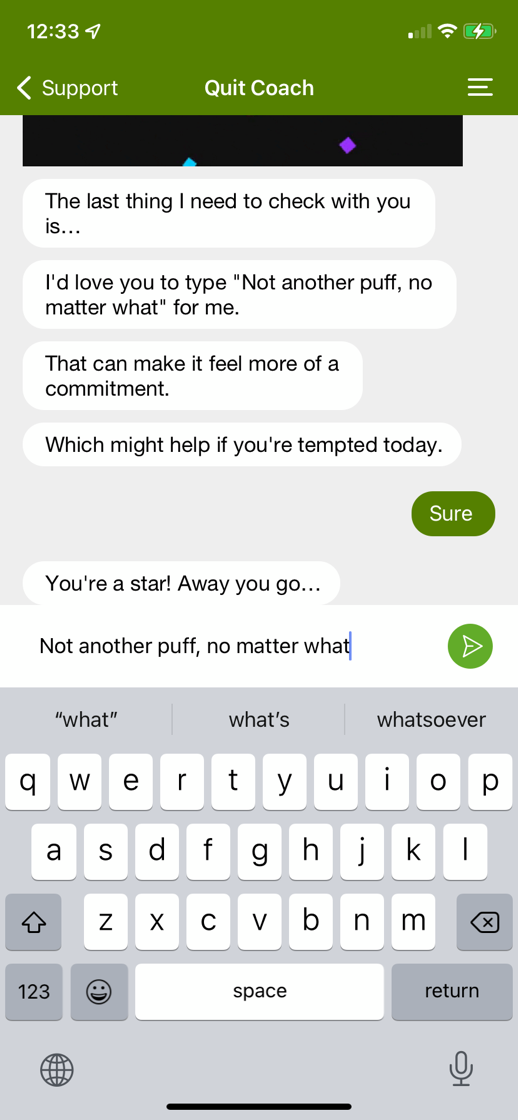

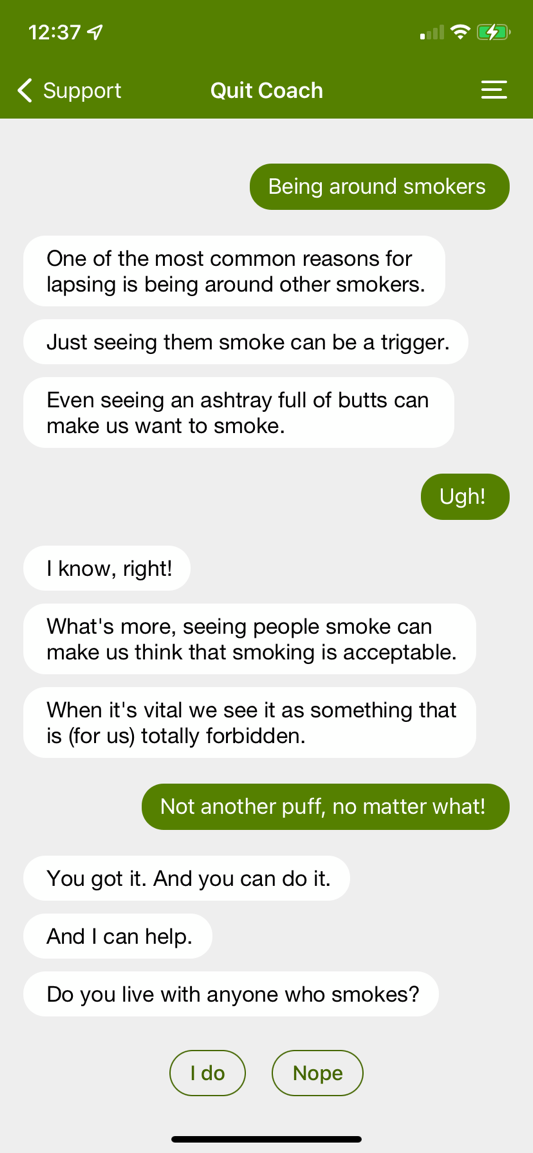

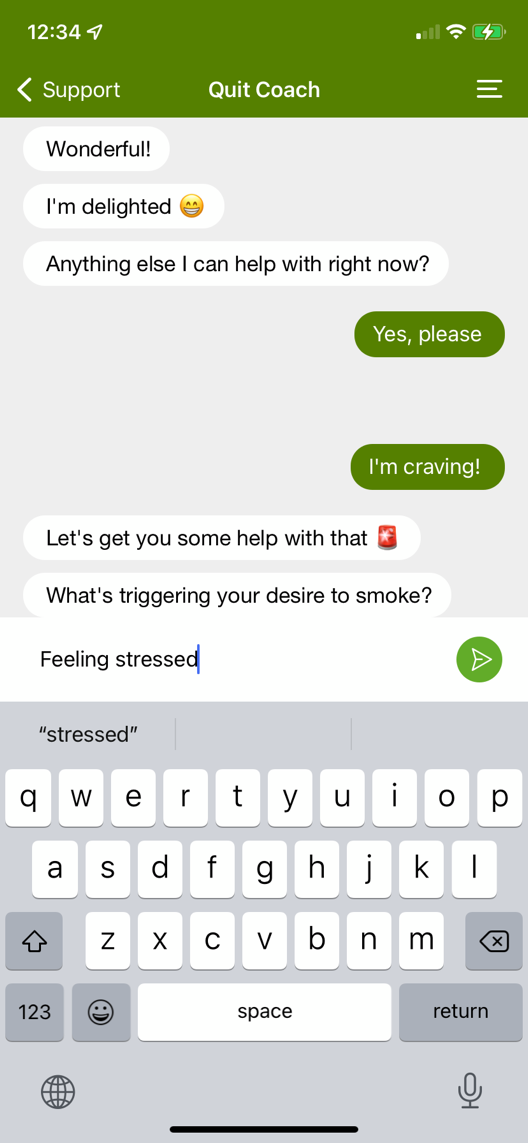


Additional participant quotations

| **Theme** | **Additional representative quotations** |
| --- | --- |
| ***Anthropomorphism of and accountability to the Quit Coach*** | ‘however much that robot is not real... it does feel more human and more like a conversation than just getting information’ (P2, 2020, Quit)  ‘I wasn’t expecting this kind of, this nature of conversation with a chat bot’ (P7, 2021, Cut Down)  ‘if one takes it seriously, people feel accountable to that’ (P12, 2021, Cut Down)  ‘you kind of developed I think I said before a respect for it almost as an entity rather than as this kind of bots’ (P13, 2021, Quit)  ‘so obviously all your friends and family do support you – but it’s nice just having that someone twice a day, just going “yep well done, keep going”’ (P3, 2020, Quit)  ‘it just felt very supportive, like, it was like a best friend, like talking to you. But also like you were talking to a psychologist....they really motivate you, when, made you feel positive, like, like proud of yourself...it was like a perfect best friend’ (P5, 2020, Quit)  ‘it worked for me because it was like talking to someone I knew, not someone who can judge me who doesn’t really know me’ (P11, 2021, Cut Down)  sometimes the app would tell me ‘oh it’s hard today, that’s normal, it’s the second day’ and I liked when he said that (P7, 2021, Cut Down)  the relationship part is very helpful. It got the information in depth, the sources are genuine, in general trustworthy. It is very cooperative, very friendly. Yeah there is definitely a bond (P12, 2021, Cut Down)  ‘I felt like found like I wanted to go in and be like look, I haven't smoked since the last time and haven’t had any cravings either like it's all going well… I weirdly felt bad going to it and saying I've had three cravings today’ (P6, 2021, Quit)  [I was motivated to engage if] I told him that I didn’t give in to a craving… [did you feel accountable?] A little yeah. I mean obviously I know it’s an app, but yeah I guess so in a way (P7, 2021, Cut Down)  ‘I chose not to [report smoking] when I did smoke and I would be like no I’m not going to send you a message. And when he checked on me, I said that I didn’t smoke but I had… because I knew I was going to smoke but I didn’t want to hear that I shouldn’t’ (P8, 2021, Cut Down) |
| ***The Quit Coach’s interaction style and format*** | ‘[GIFs] I think that was a good feature of it because I think it helps develop the relationship, which is a good thing because it means you want to keep coming back… there was a certain level of wanting to go back and get those little gifs or whatever… the positive reports I was always glad to go and have a check in like at the end of the day because I knew that it was going to be positive’ (P6, 2021, Quit)  ‘yeah the GIFs, it's interesting you mentioned that I thought the gifts may it gave it kind of an element of personality OK some of them were quite amusing actually also I think it can be hard sometimes to convey your tone or any sort of body language you know when you type something so it did I think the gifts and the emojis and stuff like that did help to give it a bit of character, bit of a personality’ (P13, 2021, Quit)  ‘I loved how positive the reinforcement was, that was fantastic. It really encourages you to do better…it was always very positive, when it said “have you had a smoke today?” and I’m like “no I haven’t and it’s like “brilliant fantastic that’s really well done you’re another step closer to this, this is getting better and things like that” so I found that was very rewarding to hear that’’ (P3, 2020, Quit)  ‘definitely the word positive reinforcement comes to mind, it kind of reminds you about certain particular things… yeah it encourages you well definitely. Uhm it gives you that sort of positive, that mantra quite a lot in the mornings and tryna keep saying ‘well done’ and ‘don’t smoke’ and don’t do it today whatever’ (P1, 2020, Quit)  ‘it was just so positive, it just made me feel very positive about everything…Adorable, it was adorable…it was very light and positive…I actually looked forward to it, because of the funny gifs and yeah…I think [it keeps you] to going, to keep your will power up, to keep you positive, to focus you more on the positive aspects of quitting smoking’ (P5, 2020, Quit)  ‘it hasn’t like taken over my life.... I think it wasn't like it didn't take up more than like a minute, minute and a half tops. Like if you answered questions in the negative it would probably take another 10-15 seconds to work through that or whatever, but if it popped up and I was just talking about 1 craving, it would only be like a minute, so it was definitely worth my time doing that’ (P6, 2021, Quit)  ‘99% of the time that was fine it was only that kind of 1% of time you’re like “sod off I’m busy”’ (P4, 2020, Quit)  ‘it was a bit long to engage. So it would be ‘are you available’ yes, ‘are you ok’ yes – so we had a few questions which were always the same which took some time to get into the real subject’ (P7, 2021, Cut Down)  ‘60-70% of the time I would check in immediately. Whenever I’m busy… [like]when I am busy with work…. you know I used to check in but after one or two hours’ (P12, 2021, Cut Down)  ‘it gives you like a quote, and then just some kind of motivational thing, and it wants you to type in this kind of mantra “not one puff not matter what” and then you kind of get on with your day’ (P1, 2020, Quit)  ‘You realise it – the robot – doesn’t really understand what you are saying and so it takes a long time to say what I was feeling… it’s sometimes just too long’ (P8, 2021, Cut Down)  ‘it was a bit long to engage. So it would be ‘are you available’ yes, ‘are you ok’ yes – so we had a few questions which were always the same which took some time to get into the real subject’ (P7, 2021, Cut Down)  it was the same everyday...I can’t remember what it was but it was like ‘oh change the record’…it’s a bit boring listening to the same thing every time (P4, 2020, Quit)  ‘sometimes I was just saying ‘yeah ok’ so he could continue with what he was saying’ (P7, 2021, Cut Down)  ‘it’s just like how are you, did you have any cravings? No? No, great. So, you don’t really want to go and say these things so sometimes I just didn’t answer because I was like ‘ok, same’... when you say ‘I didn’t smoke’ it’s getting a bit boring because it’s lots of times the same sentence and really short’ (P8, 2021, Cut Down)  ‘That at some point you just wanna go through his questions so really it doesn’t matter anymore if you say yes or no because I know where it ends and it always ends the same way’ (P11, 2021, Cut Down) |
| ***Users’ perceived need for support*** | ‘[after dinner] I would probably have a cigarette [but] the chatbot popped up instead...in that minute [of QC interaction] I could be having half a cigarette, but instead I was just having a chat with the chat bot which is really helpful…I just wanted to distract my mind [from] smoking and the chatbot was really good for that’ (P6, 2021, Quit)  ‘[I would reach for it] every craving, every time I want to smoke I will go to the app and send a message’ (P8, 2021, Cut Down)  ‘[I opened it] whenever I felt like having a cigarette I guess…[engagement was driven by]: just a bit of a distraction… I think the app engaged me enough to stop me from immediately going to get a cigarette’ (P10, 2021, Cut Down)  ‘For me it was very much a great thing to keep my hands [and] just give me time to let the craving pass [without] focusing on it…’ (P2, 2020, Quit)  ‘If you can just distract yourself and do something else you’ll forget about it [the craving] and I had that in the back of my head as well just thinking “I really really want a fag but a couple of minutes and I won’t want one” so just tried to distract myself for a few minutes and that helped’ (P3, 2020, Quit)  ‘I think certainly in the like the early, maybe the first week I reached for it more than in the second week. I think the second week I still went on it, mainly 'cause it was prompting me to go on it, but that might be a symptom of my cravings reducing after the first few days to be honest… in terms of the cravings [going] down, I felt like I maybe didn't need to use the app anymore’ (P6, 2021, Quit)  ‘Maybe for the first week it is cool to have a message everyday bc it is really hard’ (P8, 2021, Cut Down)  it kind of dwindles down to kind of extreme need, its more about the check-ins in the morning… chatbot doesn’t have much use now it’s like er ten weeks or something since I stopped (P2, 2020, Quit)  ‘if I’m honest I haven’t used it much the last couple of weeks, but at the beginning I found it really really helpful…there is like a natural, its very intense in the first week’ (P3, 2020, Quit)  ‘[as time went on] it wasn't so much you know you've done you've done so well except it was for me at that point more looking too let's not mess this up now kind of thing… I feel once you hit maybe two weeks or more it was longer the case if I just had a cigarette the other day so it's not the end of the world if I smoke I think at that point it is then you have now quit and you should not you know get back into it because you will start again at least that's my mindset after a couple of weeks’ (P13, 2021, Quit)  ‘[when it] went to 2-3 days… I [felt] like I relied on it a lot, so thought that it should be daily for a bit longer, maybe another couple of months’ (P5, 2020, Quit) |
